# Let Them Be the Judge of That: Bias Cascade in Elite Dressage Judging

**DOI:** 10.3390/ani13172797

**Published:** 2023-09-03

**Authors:** Inga Wolframm

**Affiliations:** Applied Research Centre, Van Hall Larenstein University of Applied Sciences, Larensteinselaan 26-A, 6882 CT Velp, The Netherlands; inga.wolframm@hvhl.nl

**Keywords:** equestrianism, performance evaluation, judging bias, dressage judging, systematic errors, equine welfare

## Abstract

**Simple Summary:**

Most aesthetic sports where judges subjectively rate performances can suffer from issues like systematic errors due to biases. Equestrian dressage is defined through the intricate interaction between horse and rider. Assessing these interactions can be particularly complex, often exceeding human processing capabilities. The study focuses on whether the current dressage system predisposes international judges to using biases, and inadvertently favours certain horse-rider combinations. The study examined 510 judging scores, gathered from seven elite-level dressage competitions held between May 2022 and April 2023. The effect of different factors, such as whether riders competed in their home country, if they had the same nationality as the judges, their starting order during the competition, and how they were ranked according to previous performances were analysed. Results showed that all these factors influence the final dressage results. In order to assist judges in providing objective, transparent scores, a clear evidence-based set of judging guidelines should be developed, which would prevent judges from having to resort to cognitive short cuts. That way, the complexity of judging is reduced, making scores more objective, transparent and fair.

**Abstract:**

Sport performances judged subjectively often suffer from systematic errors due to biases, with the sport of equestrian dressage being no exception. This study examines whether international dressage judges display systematic errors while evaluating elite horse-rider combinations. Data from seven 5* Grand Prix dressage events between May 2022 and April 2023 were analyzed (510 judges’ scores) using Multivariable Linear Regression Analysis. Five predictor variables—Home, Same Nationality, Compatriot, FEI Ranking and Starting Order—were studied in relation to Total Dressage Score (TS). The model accounted for 44.1% of TS variance; FEI Ranking, Starting Order, Compatriot, Same Nationality, and Home were statistically significant (*p* < 0.001). Judges exhibited nationalistic and patriotism-by-proxy biases, awarding significantly higher scores to riders from their countries (*p* < 0.001). FEI Ranking and Starting Order also influenced scores significantly (*p* < 0.001). These biases, combined, created a cascade effect benefiting a specific group of riders. To address this, measures should be taken to develop a more objective judging system that is based on unequivocal, transparent and evidence-based criteria and supports the continuous development of a fair, sustainable, equine welfare orientated sport that fosters societal acceptance,

## 1. Introduction

To this day, performance evaluation is an integral part of sport, whether at the grassroots or elite level. In nearly one-third of all sports registered with the International Olympic Committee, performance is evaluated in whole or in part with the help of referees or judges [1]. Team sports such as football, rugby and field hockey rely on referees to enforce the rules of the game, while primarily aesthetic sports such as gymnastics, ice skating, and several equestrian disciplines, depend on judges to evaluate athletic performance on the basis of predefined sport-specific criteria [2,3]. Despite the fact that officials generally receive extensive training, judging remains a highly complex task that frequently exceeds the limited cognitive capacity of humans – judges – to process information [4,5,6]. As a result, humans – judges – frequently rely on cognitive short cuts, simplified decision-making processes based on readily available, salient informational cues [7,8]. While these types of short cut can produce accurate results [9], in highly complex situations such as performance judgements in sport, they commonly lead to systematic errors in decision making, also referred to as biases or heuristics [10,11,12].

Perhaps one of the best known biases is the so-called national bias or patriotism effect, whereby judges either favour athletes from their own country or mark down athletes from other countries (e.g., [11,13,14,15]). Other types of systematic error frequently shown to be present in sports refer to the tendency of judges to rank athletes higher or lower depending on the order in which they perform (e.g., [16,17,18,19]), or the ‘reputation effect’ whereby the assessment of athletic performance is influenced by—as the name would suggest—the reputation of the athlete [20]. Similarly, the ‘memory effect’ refers to the tendency to be influenced by past performances of the same athlete [21,22]. Last, but not least, the ‘conformity effect’ describes the effect of a judge adjusting a score to align with that of fellow judges [23,24]. Even though performance-based consensus among judges is what sporting federations aim for, research has repeatedly demonstrated that judges are likely to change their scores once they are aware of the scores given by other members of the judging panel [24,25,26].

The social cognition framework, as proposed by Greifeneder et al. [27], sheds light on the processes underpinning human judgement behaviour, and may also be used to describe the principles behind judgements in a sporting context [12,28].

Prior to any type of assessment taking place, the stimulus (i.e., the element of performance) has to be perceived—in other words it must be physically seen—by the judge. Aspects that affect visual input, such as viewing angles or perspectives, have been shown to be highly influential to the judging outcome. For instance, in a study by Dallas et al [29], differences in judgement of a static element in artistic gymnastics were shown to be a function of the judges’ angle of observation. Similarly, Hüttermann et al [30] showed that erroneous assessments of the offside rule in soccer were largely attributable to the angle between players rather than the distance between the referee and the players. These findings demonstrate that an error in judgement may be due to something as apparently straightforward as where the official is positioned. 

As part of the second processing step, the perceived stimulus must be encoded and given meaning in the context of the sporting situation. Should the performance be considered excellent, below par or mediocre [31]? To that end, judges must rely on memory recall (i.e., prior knowledge and experience of the criteria or parameters employed in that particular sport) [21,22]. 

In a final step, all the information gathered until that point needs to be integrated into a cohesive whole that can be expressed in a score or final judgement. As noted by Plessner et al. [28], most judgements are formed on the basis of information derived from the environment as well as factors unique to the individual judge, such as accumulated knowledge, experience, or even aspects relating to personality or mood states [32]. The systematic errors commonly seen in judging in different sports may therefore be due to one or several judging biases that can occur in any (or all) of the three processing steps. In fact, the complexity of the judging process, combined with the speed at which a decision has to be taken and the fact that judges get to see each movement only once goes a long way towards explaining why judging is such a cognitively challenging task. 

In equestrian dressage, the idea of bias in judging is not new, with several studies demonstrating the existence of different types of systematic error [31,33,34,35,36]. As a multi-species activity, equestrianism in general is defined through the interaction between horses and humans [37,38]. Competitive success, therefore, might be argued to depend on the quality of the horse-rider interaction at the time of competition. In aesthetic equestrian disciplines such as dressage, the decision on what may be considered superior quality on the day rests with one or several judges. To aid judges in their tasks, the international governing body, the Fédération Equestre Internationale (FEI), has developed extensive guidelines on how to assess horse-rider performances. These are outlined in the FEI Dressage Handbook—Guidelines for Judging [39]. These guidelines are based on the so-called classical principles of dressage, commonly referred to as the Training Scale [39]. The six interdependent and progressive criteria propose to develop a horse’s natural physical and mental aptitudes. ‘Rhythm’ describes the regularity of each pace, in a constant tempo. ‘Suppleness’ captures the smoothness of the horse’s movement in all the different exercises and in relation to the rider’s aids. The soft, steady connection between the rider’s hand and the horse’s mouth is expressed through the term ‘Contact’, while ‘Impulsion’ outlines how the horse uses its energy in a controlled manner to propel itself forward. ‘Straightness’ refers to the alignment of the horse’s hindquarters with its forehand, on straight lines as well as on the curved track. Lastly, ‘Collection’ describes the increased activity of the hindlegs and a lowering of the hindquarters, creating an apparent lightness of the forehand. While none of the criteria refer to the welfare of the horse directly, the overarching guidelines of the FEI stipulate that equine welfare must be at the core of all riding and training of horses [40,41,42]. 

In competition, horse-rider combinations are required to demonstrate their level of training by performing different movements in quick succession, with the difficulty of movements depending on the level of training of the combination. According to the FEI Dressage Handbook [39], for each of these movements judges are required to assess the level at which a horse complies with the ideal of each of the six elements of the Training Scale, as well as the accuracy of the figure and the level of obedience of the horse (i.e., whether the horse complies willingly with the aids of the rider). These deliberations are then captured in a single mark per movement, ranging on a scale from 0 (=not performed) to 10 (=excellent) [39]. In order to compute a mark, judges are supposed to “have a clear picture in their mind as to what each mark ‘looks like’, this together with saying the corresponding words for a mark e.g., very good (9), fairly bad (3), sufficient (5), etc. helps the judge to use the scale consistently” [43]. In practice, this means that judges must be able to weigh up the relative importance of each of the different criteria prior to deciding on a score. Does the criterion of impulsion, for example, weigh more or less heavily than the criterion of contact? Does this—or should this—change per movement and perhaps even from one horse to the next, considering the morphological differences of horses? To what extent does the execution of each movement support or put at risk the welfare of the horse? 

The immense complexity of having to judge a variety of movements, across different gaits, and in quick succession constitutes a high cognitive load which, if not managed effectively, is highly likely to exceed human processing capacity [44]. Consequently, dressage judges are more likely to rely on much simpler cognitive strategies when coming to a decision, resulting in systematic judging error when judging dressage competitions [11,33,35,36].

Attempts have been made over the past few years to improve the reliability of the scores, such as the introduction of the Judges Supervisory Panel [45], the introduction of half marks (e.g., 6.5, 7.5), expanding the judging panel from five to seven judges for major championships, the use of real time scoring [46], and the publication of additional judging guidelines to assist judges on how to score serious mistakes in a test [47]. However, the dressage scoring system has yet to undergo any substantive changes, meaning that the underlying complexity of what is demanded of judges has essentially remained unchanged. It is highly probable, therefore, that the current dressage system continues to predispose judges to inadvertently using cognitive short cuts. However, to the author’s knowledge, no recent studies have investigated this. 

The current study therefore aims to determine whether international dressage judges qualified to judge at the highest level of the sport may be prone to systematic errors when assessing elite horse-rider combinations competing at the highest level of the sport. 

## 2. Materials and Methods

### 2.1. Dressage Competitions

Data from a total of seven (N = 7) 5* Grand Prix (GP) dressage competitions, held at different venues across the world, were used. These included six CDI5* GP dressage competitions held between May 2022 and March 2023 in the USA (N = 1), Germany (N = 2), Denmark (N = 1), Sweden (N = 1) and Quatar (N = 1) and the Dressage World Cup Final in the USA in April 2023. All dressage competitions were held under the auspices of the FEI, the international governing body of equestrian sports, with all tests being ridden in a 20 m × 60 m dressage arena. For each of the six GP dressage events, five judges registered with the FEI at Level 3 or Level 4 (highest level) were appointed, seated at different positions around the perimeter of the arena. A minimum of three judges were from a different home country than the host country and of different nationalities from each other (i.e., at least four nationalities were represented among the five judges). At the dressage World Cup Final, seven FEI Level 3 or Level 4 judges from six different countries were present, also seated at different positions around the arena. 

At all seven events, horse-rider combinations performed the FEI Dressage GP test. The GP is the first test horse-rider combinations will perform during any 5* dressage competition. It consists of 33 movements, each of which is assigned a single score by each judge. Eleven of these movements have a coefficient of two, meaning that the score is counted double. In addition, there is one ‘collective’ mark, also with a coefficient of two, for general impression (which includes the harmonious presentation of the rider/horse combination, the rider’s position and seat, and the discreet and effective influence of the aids). Each test lasts approximately 6.5 minutes, with times varying somewhat between horses. At the end of the test, the scores for each movement and the collective mark awarded by each of the judges are combined into a final score per judge. The total dressage score (TS) is the average of all judges’ scores for that test, expressed as a percentage of the total marks available. 

Each movement was judged on a scale from 0 (=not performed) to 10 (=excellent). Half marks from 0.5–9.5 could be used for each movement and the collective mark. The starting order of the riders was determined by a draw in groups of five, conducted in reverse order of the FEI Dressage World Ranking for horse-rider combinations (i.e., the five horse-rider combinations with the lowest world ranking competed first, and those with the highest ranking competed last) [41]. 

Judges represented the following nationalities: The Netherlands, France, Sweden, USA, Germany, Denmark, Switzerland, Portugal, Great Britain, Russia, and Luxemburg. 

### 2.2. Data Collection

Data were collected from the FEI dressage performance dashboard [48]. The dashboard and data used in this study were publicly available, therefore no ethical approval for the study was sought. The study followed the Netherlands Code of Conduct for Research Integrity [49]. For all seven events, the following data were gathered: nationality of the rider, the official FEI ranking of the horse-rider combination at the time of the competition [50], starting order of the rider, nationality of each judge, final score per judge and TS. No official FEI ranking could be determined for two riders. Their scores were removed from the data, resulting in a total data set of 510 unique judging scores. These were distributed as follows per competition: USA GP 5* (N = 60), Germany GP 5* (N = 75 and N = 55), Denmark GP5* (N = 65), Sweden GP5* (N = 45), Quatar 5* (N = 105) and USA Dressage World Cup Final 5* (N = 105).

### 2.3. Data Analysis

Preliminary analyses were conducted to ensure no violation of normality, linearity, multicollinearity and homoscedasticity. Descriptive statistics were calculated for the five predictor variables, Home, Same Nationality, Compatriot (all in percentages), FEI Ranking and Starting Order (displaying the range for both), overall TS (mean ± SD), as well as TS moderated by the variables Home, Same Nationality and Compatriot (mean ± SD). The composition of the binary variables Home, Same Nationality and Compatriot was modelled on research by Bouwens et al. [10]. Variable Home was composed by assigning the value of 1 if the rider was competing in his/her home country and the value of 0 if s/he was competing in a country other than their home country. Variable Same Nationality comprised the value of 1 if rider and judge had the same nationality, and the value of 0 if the rider’s nationality was different from that of the judge. Variable Compatriot contained the value of 1 if the rider was a compatriot of one of the other judges on the panel, and a value of 0 if the rider’s nationality was different to that of all of the other judges. Variable FEI Ranking was modelled as a continuous variable and composed of the official rankings of the participating horse-rider combinations based on the FEI Dressage World Ranking [51]. Variable Starting Order was also modelled as a continuous variable and contained the starting position for each rider at each competition (i.e., the rider that competed first was assigned a value of 1). 

A univariable linear regression analysis was conducted initially to test the independent contribution of each of the five predictor variables on TS: Home, Same Nationality, Compatriot, FEI Ranking, and Starting Order. 

The five predictor variables were then entered into the multivariate equation simultaneously in order to explain any potential unique contribution of each variable to the final model, as well as their combined contribution as a group. Semi-partial correlation coefficients squared were calculated to determine the percentage of variability of TS uniquely accounted for by each predictor variable [52]. Statistical significance was set at *p* < 0.05.

Lastly, independent sample *t*-tests (two-tailed) were conducted to test for significant differences in TS for the variables Same Nationality, Compatriot and Home. In order to protect against a Type 1 error, a Bonferroni adjustment was applied by dividing the previous alpha level of 0.05 by 3, resulting in a new alpha level of 0.017. To determine the strength of the effect size, Cohen’s d was calculated for all tests [53]. All statistical tests were conducted using the statistical package IBM SPSS Statistics, version 28.0.1.0. 

## 3. Results

### 3.1. Initial Inspection of the Data 

Inspection of the data showed that the assumptions of normality, linearity, multicollinearity, and homoscedasticity had not been violated. Examination of Mahalanobis Distance and the Scatterplots revealed no unusual outliers [53]. Subsequent analyses revealed no further violations of the nature and distribution of the data. Tolerance values ranged from 0.627 to 0.843 and Variance Inflation Factor values from 1.187 to 1.595, indicating no issues with multicollinearity. For TS, the mean ± standard deviation SD. TS was 70.91% ± 4.13. The ranges for FEI Ranking and Starting Order were 285 (2–287) and 20 (1–21) respectively. 

### 3.2. Univariable Linear Regression Results

Independent univariable linear regression analyses of the five predictor variables on TS revealed highly significant individual contributions for FEI Ranking (beta regression coefficient = −0.566; *p* < 0.001), followed by Compatriot (beta regression coefficient = 0.421; *p* < 0.001), Starting Order (beta regression coefficient = 0.264; *p* < 0.001), Same Nationality (beta regression coefficient = 0.208; *p* < 0.001) and Home (beta regression coefficient = 0.131; *p* = 0.003) (see Table 1). All five predictor variables were subsequently entered into the multivariable linear regression model. 

### 3.3. Multivariable Linear Regression Model

Multivariable linear regression analysis determining the effect of the five independent variables Home, Same Nationality, Compatriot, FEI Ranking, and Starting Order on TS revealed that 44.1% of the variance in TS could be explained by the model as a whole, with R Square = 0.441 (F(5,504) = 79.484; *p* < 0.001). All five predictor variables (FEI Ranking, Starting Order, Compatriot, Same Nationality, and Home) were statistically significant (*p* < 0.001 for all; see Table 2).

Comparing the individual contributions of each predictor variable in the multivariable model, the strongest individual contribution to TS was Compatriot (beta regression coefficient = 0.403; *p* < 0.001), followed by FEI Ranking (beta regression coefficient = −0.343; *p* < 0.001), Same Nationality (beta regression coefficient = 0.287; *p* < 0.001), Home (beta regression coefficient = −0.136; *p* < 0.001), and Starting Order (beta regression coefficient = 0.133; *p* < 0.001). 

Individual contributions of each predictor variable to Total R Square were calculated using the squared value of the semi-partial correlation coefficients: Compatriot = 0.319; FEI Ranking = −0.280; Same Nationality = 0.240, Starting Order = 0.122 and Home = −0.117. The unique contributions to the total variance of TS expressed as percentages were Compatriot = 10.18%; FEI Ranking = 7.84%; Same Nationality = 5.76%, Starting Order =1.49% and Home = 1.37%. These data also indicate how much the total variance would decrease if one of these variables was removed [52]. 

### 3.4. Tests of Differences

Independent sample t-tests revealed highly significant differences in TS for Home (t(508) = −2.969; *p* = 0.003), Same Nationality (t(508) = −4.803; *p* < 0.001), and Compatriot (t(445.05) = −10.314; *p* < 0.001) (Table 3). 

## 4. Discussion

The current study aimed to investigate whether highly qualified dressage judges may be prone to systematic errors (i.e., judging bias) when assessing elite horse-rider combinations. The current results confirm that, at the highest level of international dressage, dressage scores are not simply an objective reflection of horse-rider performances on the day. Almost half of the variance in dressage scores can be explained by the combination of other factors: whether judges and riders share the same nationality, previous performance as indicated by FEI ranking, starting position, or whether the rider is competing in their home country. 

### 4.1. Nationalistic Bias, Patriotism by Proxy and Home Advantage

The effect of nationality on judging behaviour has been well-documented in other sports [11,14,54]. As demonstrated in the current study, dressage judges do not seem to be immune from this, systematically giving higher scores to riders who share their nationality. With an individual contribution to the total variance in scores of 5.76%, the effect of the nationalistic bias might be considered relatively small yet is highly significant. What is more, judges also tend to give higher scores to riders who are of the same nationality as one of the other members of the judging panel. Here, the individual contribution of this indirect nationalistic bias, or “patriotism by proxy” bias is almost twice as high, namely 10.18%. Logic dictates that any rider who benefits from the nationalistic bias of one of the judges also benefits from the ‘patriotism by proxy’ bias from all other judges. As a result, riders with the same nationality as any one of the judges on the panel might receive scores that are, on average, 3.54% higher than riders with a different nationality. 

Interestingly, statistical results suggest that, when considering the variable “Home” independently, riders competing in their home country would be at a slight disadvantage, indicated through the negative direction of the semi-partial correlation and the (relatively small) individual contribution of 1.37% to the total variance of TS. However, while it might, in theory, be possible that there is no judge of the host nation represented in the jury panel, in practice this is seldom the case, and indeed, in the seven competitions analysed for this paper, a judge from the host nations was part of every judging panel at every competition. Thus, the disadvantage for riders competing at home will be cancelled out by the advantages of the nationalistic and patriotism by proxy biases, resulting in a cumulative advantage of 1.19% (as depicted in Table 3). 

Since the so called “home advantage” has been demonstrated in a variety of sports [55], including soccer [56], cricket [57], boxing [58] and gymnastics [59], the question presents itself why dressage judges would (inadvertently) disadvantage riders competing on home turf. According to the FEI rules of dressage, nationalistic judging is considered a conflict of interest and must be declared as such by officiating judges [41]. It is possible, therefore, that judges make an active effort to refrain from being nationalistic in their judging, thus overcompensating when assessing home competitors. Nevertheless, any deliberate attempt to be stricter is eventually cancelled out by the judges’ subconscious adherence to in-group norms. 

Most notably, the differences in scores caused by nationalistic and patriotism by proxy biases may impact considerably on the final rankings of a competition, as evidenced by the significant differences in scores for riders who are from the same country as the judges on the judging panel and those who are not (see Table 3). To illustrate what this might mean in practice: at the Dressage World Cup Final in 2023, the competitor who placed 7th held a different nationality than any of the judges. With an additional 3.54%, she would have ended up in 5th place. 

Current findings mirror results from Sandberg [36] who found significant evidence for nationalistic and patriotism by proxy biases. Sandberg argued that these systematic errors can best be explained by judges creating a temporary ‘in-group’. One of the concepts considered central to in-group favouritism is that of social identity [60,61] (i.e., the idea that a person’s self-concept is, at least in part, determined by their membership of a social group). In order to maintain a positive social identity, members of that group are likely to exhibit prosocial behaviours towards other members of their group, for example by demonstrating reciprocal support or ensuring that the entire group derives some kind of benefits [62]. At high level dressage competitions, such temporary in-groups might include individuals (i.e., other judges) who share the same relevant characteristics (judging status) and experiences (having been at a number of international events before). What is more, an in-group is also likely to include, even if only at the periphery, individuals (i.e., riders) who share the same ethnic background. After all, nationality has consistently been identified as one of the primary identifiers for in-group favouritism [63,64]. 

As a result, whenever riders who hold the same nationality as one of the members of the judging panel enter the ring, judges will, unconsciously, be inclined to favour them over others. This type of in-group favouritism has been demonstrated time and again within a sporting context [65] as well as in other settings such as work-related performance reviews [66] or court-room decisions [67]. 

### 4.2. FEI Ranking & Starting Order

In-group favouritism through nationalistic types of biases is not only inherently unfair [68], but it may also have unintended additional side effects. Since all international results are registered with the FEI, a higher or lower final placing at an international competition will invariably result in a higher or lower FEI ranking. As current findings show, FEI Ranking also has a significant effect on the final score, with a unique contribution of 7.84%. At first glance, these results may not seem altogether surprising. After all, across the equestrian community, the FEI World Ranking List has long been considered a reliable indicator of horse-rider ability and a lower numerical number in the ranking (i.e., a 3rd, 2nd or 1st place) intuitively correspond to higher scores. This relationship is indeed reflected through the negative direction of the beta regression coefficient (see also Table 2) As such, FEI ranking inherently contributes towards rider reputation (e.g., [69,70,71,72]). Research in a myriad of fields, such as business management [73,74], customer retention [73], as well as sports [20,75] has shown reputation to be a highly important moderator of assessment behaviour: the greater the reputation, the more likely a positive assessment. What is more, one of the core principles of social cognition research dictates that easily accessible information is more likely to influence judgement [76], in particular at times of high cognitive load [77]. Therefore, the greater a rider’s reputation, the more likely it is that judges will – inadvertently – be drawing on information related to that rider’s past performances rather than current form.

Finally, FEI Ranking also has a direct effect on the starting order of competitors, with lower ranked riders having to compete earlier in the day. Current findings demonstrate a relatively small, yet significant individual contribution of Starting Order on the final score. While the current practice of determining the starting order by draw in groups of five might have gone some way towards mitigating the well-known order bias [16,18], riders competing later in the day will, on average, continue to benefit. The recency effect dictates that in situations of high cognitive load, decision makers will overly rely on the information presented last to make a decision (e.g., [44,78]). Research has indicated that comparisons in one direction lead to overestimation of “novel” features in any new performance. Seeing that, at an elite level, horse-rider combinations generally have more unique positive features, riders competing later in the day are likely to receive higher scores [79]. What is more, as has been shown by Arnold et al. [77], highly experienced individuals do not seem to be protected from this type of bias, but may even be more prone to it than their less experienced counterparts. As such, the current practice of letting higher ranked combinations start later in the day is likely to predispose judges towards engaging in an – inadvertent – order bias.

### 4.3. Time for Change

Elite dressage competitions may be considered the showpieces of the sport. What is more, while elite riders may be considered the paragons of equestrian sport, judges should be viewed as the guardians of its core principles. At present, however, judges are being asked to do the impossible: they have to make lightning fast decisions, time and again, for every individual movement performed, while having to take into account a vast number of performance indicators, ranging from a horse’s natural athletic ability, the standard of training across all aspects of the Training Scale, levels of accuracy in the arena as well as the quality of the horse-rider relationship as evidenced through levels of obedience, all the while ensuring that the welfare of the horse is guaranteed throughout [42]. In response to such cognitive complexities, judges invariably and inadvertently have to draw on other, more readily available sources of information, such as nationality, previous performance, reputation and starting order. As evidenced by current findings as well as previous research [11,33,36] these cognitive short cuts subsequently combine to form a veritable ‘bias cascade’, whereby one type of bias enhances the effect of another. While such a system may end up being advantageous for the more established horse-rider combinations from countries that provide judges at elite competitions, it is neither fair to the judges nor does it support the development of dressage as a transparent, objective, and sustainable sport that prioritises the welfare of, and fairness to, its human and equine athletes. 

It is time, therefore, that efforts are taken to develop a more transparent, unequivocal judging system that relies on evidence-based criteria, in order to pave the way for a more accurate, reliable, and objective way of judging. A division of tasks among judges, for example, would likely lower the cognitive load for each of them, thus decreasing the likelihood of over-reliance on cognitive short cuts. Consideration could be given to dividing the tasks into equine welfare signals, athletic performance, rider effectiveness, and accuracy of movement. All tasks should be evidence-based and be open to continuous review. 

### 4.4. Limitations

The current study assessed dressage scores of seven elite level dressage competitions which, even though statistically sound, may be considered a relatively small sample size. It could, therefore, be argued that the systematic errors identified in the analysis might merely be a reflection of superior riding skills of riders from countries who are also able to appoint high level judges and that international dressage has traditionally been dominated by a limited number of nations. As a result, these nations would then be more likely to have in place an advanced sporting infrastructure, which may predispose them towards producing, on average, superior horses and horse-rider combination. However, such an argument may be countered by the findings of the two extensive studies by Heiniger & Mercier [11] and Sandberg [36]. Both studies had large sample sizes and reported findings that mirror those of the current study, supporting the existence of systematic error due to different types of unconscious bias. What is more, equestrianism has developed into a highly international sport [80], supported by the dynamic trade of horses and trainers, as well as entire training systems [81]. The emergence of talented riders from across the world should therefore be both possible and likely – assuming, of course, that such talent is recognised and rewarded at international shows through objective, non-biased judging.

## 5. Conclusions

The present study provides evidence of a number of systematic biases in the judging of elite horse-rider combinations in dressage competitions. Notably, judges showed nationalistic bias and patriotism by proxy, favouring riders from their own country and those of their fellow judges. The benefits of these cognitive short cuts can even go as far as cancelling out any of the disadvantages riders might otherwise experience when competing at home. Moreover, the study revealed that FEI ranking of the horse-rider combinations as well as the starting order significantly influenced the final scores.

These biases form what may be referred to as a bias cascade, which is likely to lead to inadvertent, yet nonetheless unfair, advantages for certain riders. The findings of this study align with previous research, reinforcing the importance of addressing systematic errors in judging in the sport of dressage.

The study emphasizes the need for a more transparent and objective judging system in dressage to safeguard principles of fair and equitable sporting standards. The current system’s inadvertent reliance on cognitive short cuts and biases poses risks to the integrity and sustainable development of equestrian dressage, which may also undermine its social acceptance. 

To address these issues, the FEI should prioritize the development of clearly defined, unequivocal judging guidelines that are based on evidence-based equine welfare-orientated criteria. This would enable judges to assess performances more accurately and impartially, leading to a more accountable sport with equine welfare at its core. By implementing a more objective and transparent judging system, the sport can better ensure the welfare of horses and maintain its social license to operate.

## Figures and Tables

**Table 1:** Independent univariable linear regression analyses of five individual predictor variables on TS, based on 510 unique judging observations at seven 5* GP dressage competitions.

Predictor Variable	Unstandardized Coefficients		Beta Regression Coefficients	t	Sig.	95% Confidence Interval		Semi Partial Correlation Coefficients	R-Square
	B	Std. Error				Lower Bound	Upper Bound		
FEI Ranking	−0.039	0.003	−0.566	−15.482	<0.001	−0.044	−0.034	−0.566	0.321
Starting Order	0.231	0.038	0.264	6.168	<0.001	0.158	0.305	0.264	0.070
Home	1.189	0.400	0.131	2.969	=0.003	0.402	1.975	0.131	0.017
Same Nationality	2.487	0.518	0.208	4.803	<0.001	1.470	3.504	0.208	0.043
Compatriot	3.531	0.337	0.421	10.472	<0.001	2.869	4.194	0.421	0.178

**Table 2:** Multivariable linear regression model of the five predictor variables on TS, based on 510 unique judging observations at seven 5* GP dressage competitions.

Predictor Variable	Unstandardized Coefficients		Beta Regression Coefficients	t	Sig.	95% Confidence Interval		Semi Partial Correlation Coefficients	Total R-Square
	B	Std. Error				Lower Bound	Upper Bound		
FEI Ranking	−0.024	0.003	−0.343	−8.415	<0.001	−0.029	−0.018	−0.280	
Starting Order	0.116	0.032	0.133	3.652	<0.001	0.054	0.179	0.122	
Home	−1.242	0.354	−0.136	−3.504	<0.001	−1.938	−0.546	−0.117	
Same Nationality	3.429	0.475	0.287	7.220	<0.001	2.496	4.362	0.240	
Compatriot	3.374	0.353	0.403	9.570	<0.001	2.681	4.067	0.319	
Total MLRM									0.441

Total MLRM, Total multivariable linear regression model.

**Table 3:** Mean TS scores according to binary predictor variables with “yes” confirming that the athlete complied with the conditions as set out in the predictor variable. *p*-values were set using a Bonferroni adjustment (*p* = 0.017).

Predictor Variable	Yes	No	Difference	*p*-Value	Cohen’s d
	TS score (mean ± SD) (Distribution of scores between the two conditions)				
Home	71.77% ± 4.07%	70.58% ± 4.15%	1.19%	=0.003	0.29 (small effect)
	(N = 151; 29%)	(N = 359; 71%)			
Same Nationality	73.075 ± 3.58	70.58% ± 4.12%	2.49%	<0.001	0.61 (medium effect)
	(N = 72; 14.11%)	(N = 438; 85.88%)			
Compatriot	72.93% ± 4.02%	69.39% ± 3.57%	3.54%	<0.001	0.94 (large effect)
	(N = 222; 43.53%)	N = 288; 56.47%			

SD, standard deviation; TS, Total Dressage Score.

## Data Availability

Publicly available datasets were analyzed in this study. This data can be found here: https://performancedashboard.fei.org/ (accessed on 31 August 2023).
